# Therapeutic Vaccines and Cancer Immunotherapy

**DOI:** 10.3390/vaccines8040596

**Published:** 2020-10-10

**Authors:** Darshak Bhatt, Toos Daemen

**Affiliations:** Department of Medical Microbiology and Infection Prevention, University Medical Center Groningen, University of Groningen, PO Box 30 001, HPC EB88, 9700RB Groningen, The Netherlands; d.bhatt@umcg.nl

## 1. Introduction

Cancer immunotherapy and immunization are the next steps towards safe and effective cancer treatment. Checkpoint blockade therapy, tumor-specific antibodies, adoptive cell therapies, viral vectors for gene therapy or oncolysis, and diverse platforms of tumor vaccines are being applied in clinics or are being tested in various stages of pre-clinical and clinical research.

In contrast to the prophylactic human papillomavirus (HPV) cancer vaccines, therapeutic vaccines are meant for the treatment of cancer. While the prophylactic HPV vaccination aims at the induction of virus-specific antibodies, therapeutic immunization aims at inducing tumor-specific adaptive immune responses. To date, the therapeutic efficacy of most therapeutic cancer vaccines, in general, is lower than expected, as based on preclinical studies. This limited clinical response may, amongst other reasons, be due to (i) insufficient or inappropriate activation of antigen-specific immune effector cells in cancer patients caused by immune tolerance or immune suppression, (ii) the immune suppressive tumor microenvironment, (iii) limited homing and accumulation of immune effector cells into the tumor, and (iv) limitations due to narrow-spectrum and highly specific adaptive immune responses, which do not target the whole heterogeneous population of tumor cells [1,2]. Thus, research on further improvements to pre-emptively increase the chance of therapeutic efficacy of cancer vaccines in the clinic is still essential.

To this end, this issue focuses on providing a better understanding of the variables governing the disease state of the patients, which in turn will reveal molecular targets that can be considered for a strategic therapeutic design aimed at better clinical outcomes. Moreover, in this issue we learn how individual statuses of the patients may have an impact of the clinical outcome mediated by therapeutic vaccines or checkpoint therapy, thus demanding strategies that can be applied for personalized therapy.

## 2. Cancer: Understanding the Disease and Spotting Strategic Targets for Therapeutic Immunization

Cancer is a diverse and complex disease caused by specific changes in the genome that contribute to cell transformation, and therefore requires proper classification based on molecular and genetic characteristics. The review by Tuyaerts and Amant gives an insight into endometrial stromal sarcomas (ESS) by first providing an overview of the clinicopathological features of low-grade and high-grade ESS based on molecular markers [3]. Despite these molecular differences, the authors indicate that a common feature of both low-grade and high-grade ESS is recurrent chromosomal translocations that may result in the fusion of genes involved in regulatory processes driving neoplastic transformation. They argue that these translocation–fusion proteins can represent potent tumor antigens as they are tumor-specific, many of these breakpoints are shared by several patients, and often required for tumorigenesis. Accordingly, a road-map to determine and screen the immunogenicity of these translocation-breakpoints in ESS is provided where prediction algorithms of peptide-HLA binding and in vitro immunogenicity assay play a crucial role in defining the target antigens for therapy.

As a practical approach to antigen selection and screening, Terbuch and Lopez write that next-generation sequencing (NGS) can aid in the identification of individual mutations and could pave the way for personalized cancer vaccines [4]. A major limitation pertaining to immunotherapy is that the efficacy is limited to cancer patients with high-mutational burden and pre-existing immune responses [5]. Screening antigens via NGS seems an interesting approach but requires extensive research before it is put to practical use. A major challenge faced by NGS these days is the low prediction accuracy of high-affinity binders by the existing algorithms, indicating that a multidisciplinary approach in combination with bioinformatics is required for functional improvisation. The authors also comment on how to improve against the challenges arising due to tumor evolution and loss of antigens, and on determining the right combinations and schedule of immunotherapy in a personalized manner.

## 3. Immune Status of Individual Patients Impact the Therapeutic Efficacy in Terms of Anti-Cancer Response

Recent literature from clinical research has demonstrated that initial patient status has a large impact on the clinical endpoints, for better or worse. For example, in checkpoint immunotherapy, it was observed that PDL1 status of patients correlates strongly with the observed efficacy and is routinely used as a biomarker to predict efficacy [6]. In a similar approach, the immunocompetent status of the patient has been studied and found to influence cell-based vaccination therapy as described in the review by Lluesma et al. in this issue [7]. Viral vector based vaccines may suffer from antibody-mediated anti-vector immune responses that impede the therapeutic outcomes. Additionally, the presence of immune cells with a regulatory phenotype prove to be a challenge in the case of stimulating a local immune response in the case of myeloid-derived suppressor cells and peripheral immune responses regulated by regulatory T cells.

To better understand such anti-inflammatory and regulatory responses mediated by the immune system, Van Ee et al. review the effect of BDCA1+CD14+ immunosuppressive cells in cancer and argue that these cells can be targeted to restore therapeutic efficacy [8]. The authors provide an insight into how the presence of BDCA1+CD14+ cells in patients may suppress the induced immune response in an antigen-specific manner systemically and at the tumor site, whereas, BDCA1+CD14+ cells in dendritic cell (DC) vaccines may directly hamper vaccine efficacy. They review the presence of BDCA1+CD14+ cells in solid cancers and their immune-suppressive functions, and evaluate the presence of BDCA1+CD14+ cells in leukemic cancers, suggesting that the presence of BDCA1+CD14+ cells correlates with clinical features of acute and chronic myeloid leukemia. The authors comment that further research in understanding the development of BDCA1+CD14+ cells is necessary to determine specific targets and improve the efficacy of dendritic-cell based vaccines. In addition, the earlier-mentioned review by Lluesma et al. dissects how observed ineffectiveness in anticancer responses is dictated by the immunocompetent status of cancer patients, supporting progression of cancer via mechanisms like immune evasion [7]. The authors argue that most of the trials apply DC vaccines to patients with an advanced gynecological or breast cancer, which may be a factor mediating low response rates in clinical settings. Alternatively, adjuvant settings of DC-based vaccination report higher response rates as compared to metastatic settings in terms of tumor-specific immunity. Ineffective stimulation of immune responses may occur due to impaired development of DCs, immune-tolerance, and defects in peripheral blood DCs of cancer patients. Thus the authors suggest the notion of vaccinating cancer patients with early-stage disease, when possible, to improve response rates, while considering screening patients and assessment prior to vaccination. Simultaneous boost via combinatorial therapy—e.g., checkpoint blockade—may improve the expected outcomes in clinics. Further indications can be gained by studies focusing on the correlation of routes of administration with objective clinical responses, and alternatively by screening for ideal vaccine vectors designed to overcome the barriers of regulatory immune responses while maintaining a safe approach to vaccination.

## 4. Strategic Vaccine Design and Combinations Thereof

Generating a vector for therapeutic vaccination requires that it should be engineered to be safe and efficacious. Thus, vector design becomes equally important to vaccine development as is epitope screening and knowledge of the target disease. The present issue brings the opinion and insight of various researchers on strategies currently used, and necessary future improvements in designing ideal delivery vectors for therapeutic vaccination. Due to advances in the field of synthetic biology, it has become possible to engineer DNA-based, RNA-based, bacterial chassis, and viral vectors for safe and effective immunization. Each vector design is intended to bring together genetic components and respective resources to function, including but not limited to the transcription-translation of antigens, appropriate presentation, and the activation of target immune cells.

One such example reviewed by Flickinger et al. in this series is of an engineered bacterial chassis—*Listeria monocytogenes*—that has demonstrated impressive therapeutic benefits in pre-clinical models and have and are being tested in clinical trials for anti-cancer therapy [9]. Attenuated *Listeria* strains have been developed via deletion or episomal replacement of virulence genes, or via photochemical-inactivation of the bacterial vectors. These strategies of attenuation have an advantage over heat-kill inactivation as they allow the vector to invade host cells and secrete target antigens in the cytosol for antigen presentation, thereby inducing CD8+ T-cell responses. Additionally, antigen-mediated antitumor responses can be enhanced via fusion with *Listeria* antigens, such as Listeriolysin-O, ActA proteins, resulting in stronger antitumor immune response. Future directions here seem to explore combination approaches using Lm vaccines with radio-/chemotherapy, checkpoint blockade, or perhaps a heterologous boost of vaccination with viral vectors re-stimulating antigen-specific immunity.

Pre-clinical and clinical research in the field of viral vectors for antigen delivery is comparatively more advanced than other (bacterial) vector-based therapies. However, various studies to date have faced multiple challenges to the application of viral vector-based vaccines in terms of anti-vector antibody responses, T cell-specific responses, and subsequent resistance to virotherapy [10,11]. Consequently, vaccine design strategies have focused on improving tumor-associated antigen-specific responses instead of anti-vector immunity. In this current issue, Chondronasiou et al. report one such an improvement in stimulating anti-melanoma T cell mediated immunity by strategic engineering of Adenoviral vectors to selectively target dendritic cells in skin and lymph nodes, and deliver melanoma antigens (MART-1) for reshaping the subsequent immune responses [12]. The authors demonstrate the possibility of an off-the-shelf DC-targeted Adenoviral therapy, made possible by a chimeric-design between serotype-5 Adenoviral capsid and serotype-3 knob protein, and has an enhanced T cell priming ability as compared to conventional infection strategies. However, the authors do indicate the possibility of pre-existing anti-adenoviral immunity that can impair therapeutic efficacy, thus requiring further research on these lines in future.

Apart from engineered microbial vaccines, cell-based immune therapies have gained increasing attention for cancer treatment. T cell-based therapies, including CAR-T cells, have been favored due to the possibility of autologous T cell generation, and persistence response against targeted epitopes selected from a myriad of tumor-associated antigens. However, technical difficulties in obtaining a large proportion of such cells from patients prove to be a major challenge these days [13]. To overcome this obstacle, Xiong et al. explored the genetical engineering of hematopoietic stem cells (HSCs) in a murine model that can be reprogrammed to develop into antigen OVA-specific T cells via cloning T cell receptors into these HSCs [14]. Of note, the authors demonstrate that such OVA-specific T cells can be effectively generated from HSC lineages and are capable of developing in vivo when combined with proinflammatory signals while maintaining their specificity to induce anti-tumor responses.

## 5. Optimizing Therapeutic Vaccination by Combinatorial Approaches and Targeted Delivery

After vector design, research is focused on strategies of vaccine delivery and combinatorial approaches to build-on the immune responses initiated by the vaccines. As an emerging strategy for targeted delivery of therapeutics, Thadi et al. describe an interesting approach of intraperitoneal immunotherapy for metastasis of peritoneum in their review [15]. They discuss how, despite of being a lethal diagnosis, peritoneal metastasis has an improved chance of positive outcomes due to targeted delivery, made possible by intraperitoneal immunotherapy. Both clinical trial results and preclinical experimental studies reviewed here have supported the notion of intraperitoneal delivery of checkpoint therapy, EpCAM targeting antibodies, engineered-NK cell-based therapies, CAR-T cells, and even virus- or dendritic cell-based therapies. In terms of combinatorial approaches to improve vaccination-mediated immune responses, the review by Dutcher and Bilen provides detailed information of pivotal trials in genitourinary malignancies utilizing cancer vaccines and checkpoint combination [16]. The authors explain how active developments in the field of vaccination for prostate, renal, and bladder cancers have shown promise in early development. However, these autologous cell-based, DNA-based, or virus-based vaccines have remained from resulting in expected clinical outcomes when applied as mono-therapies. Consequently, the authors argue that a combinatorial approach to vaccination along with checkpoint blockade seems to be a rational option, with already some pre-clinical observation supporting this statement. Future trials are awaited with high expectations, provided the established good-performance of checkpoint therapy in clinics. Such combinatorial approaches are aimed to restore the impaired immune responses due to the regulatory tumor microenvironment and can potentially boost the efficacy profile of vaccines applied prior as monotherapies.

Moreover, the efficacy of such engineered therapies also depends on features external to vaccine design and delivery strategies. Therapeutic optimization, thus, is a difficult task and requires a systemic understanding of the disease and its respective microenvironment. In cancer, such microenvironments remain in a dynamic flux of processes involving tumor cells, stromal cells, and immune cells. A diverse set of simultaneous interactions occur in such microenvironment that may result in tumor cell death, or metastasis via immune evasion [17]. Soluble signals, coupled with membrane-based protein–ligand interactions, are some of the most frequent and primary interaction studied by various research groups. In addition to these, recent interest in vesicle-mediated communication has shed some light on these complex, yet defining interaction that govern the clinical outcomes of the patients. The review by Jella et al. in this issue provides an elaborate discussion on vesicle, especially exosome biology, focusing on the role of exosomes in mediating intracellular communication and their ability in influencing the immune system of patients for better or worse outcomes [18]. The authors comment that this immunomodulatory role of exosomes in cancer can be exploited for therapy, where exosomes potentially can be engineered for delivery of therapeutics or may also serve their function as indicative biomarkers of therapeutic responses. Future research in the field of exosome biology seems promising in the development of targeted therapy by modulating exosome selectivity, in designing homogeneous populations of exosomes to decrease variability, and in developing strategies to load exosomes with antigens, nucleotide-based therapeutics, chemical drugs, or protein-based signals to improve immune responses in patients.

## 6. Conclusions

Vaccine technology has evolved to include a wide range of vectors, high-throughput methods to screen immunodominant tumor-associated antigens, combinatorial strategies, and adaptations in therapeutic administration based on individual patient requirements. However, tumor as a target also evolves constantly and participates in the Darwinian arms-race via mechanisms of clonal selection and immune evasion. With technological advances and development in bioengineering, thus it would become important to focus on evolutionary stable strategies of therapy design that are aimed at being effective despite spatiotemporal selection pressures and persist as a long-term mediator of protection against diseases like cancer.
